# The Efficacy, Safety, and Cost Benefit of Olanzapine versus Aprepitant in Highly Emetogenic Chemotherapy: A Pilot Study from South India

**DOI:** 10.1155/2016/3439707

**Published:** 2016-01-27

**Authors:** Govind Babu, Smitha Carol Saldanha, Lakshmaiah Kuntegowdanahalli Chinnagiriyappa, Linu Abraham Jacob, Suresh Babu Mallekavu, Loknatha Dasappa, Pretesh Rohan Kiran, Aparna Sreevatsa, Sandhya Appachu, Vineetha Unnikrishnan, Venugopal Arroju

**Affiliations:** ^1^Department of Medical Oncology, Kidwai Memorial Institute of Oncology, Bangalore 560030, India; ^2^Department of Community Health, St. John's Medical College, Bangalore 560034, India

## Abstract

*Background*. The efficacy, safety, and cost benefit of olanzapine (OLN) when compared to aprepitant (APR) in the prevention of chemotherapy induced nausea and vomiting (CINV) in patients receiving highly emetogenic chemotherapy (HEC) were evaluated.* Methods*. A prospective pilot study was done in chemotherapy-naive patients receiving HEC to compare OLN versus APR along with palonosetron and dexamethasone. 100 patients consented to the protocol and were randomized and evaluated for Complete Response (CR) (no emesis, no rescue).* Results*. CR was 86% for the acute period, 86% for the delayed period, and 80% for the overall period in 50 patients receiving the APD regimen. CR was 84% for the acute period, 88% for the delayed period, and 78% for the overall period for 50 patients receiving the OPD regimen. Patients without nausea were APD: 88% acute, 84% delayed, and 84% overall, and OPD: 84% acute, 88% delayed, and 84% overall. There were no significant grade 3 or 4 toxicities. OPD was comparable to APD in the control of CINV.* Conclusion*. In this study, there was no significant difference between olanzapine and aprepitant in preventing CINV with highly emetogenic chemotherapy. Olanzapine may thus be used as a potential, safe, and cost beneficial alternative to prevent nausea and vomiting in HEC.

## 1. Introduction

Chemotherapy induced nausea and vomiting (CINV) is a significant problem in oncology settings. The lack of timely intervention can result in poor compliance to subsequent chemotherapy cycles. Also added is the cost of undue hospital admissions to manage fluid and electrolyte disturbances, nutrient depletion, and other related effects [1–4]. These affect the patient's quality of life and performance status and make them vulnerable to further emesis in next cycles due to anxiety associated with bad experience. The types of emesis are acute emesis (immediate onset to 24 hrs resolution), delayed emesis (24 hrs later onset to 7 days), breakthrough emesis (despite prophylaxis), refractory emesis, and anticipatory emesis (prior to next chemotherapy) [5–9].

The NCCN currently outlines four categories of emetogenic potential for parenteral chemotherapeutics: high emetic risk (90% of patients experience acute emesis), moderate emetic risk (30 to 90%), low emetic risk (10 to 30%), and very low emetic risk (<10%) [10, 11].

Presently, the standard of antiemetic care while using highly emetogenic chemotherapy (HEC) is aprepitant/fosaprepitant with dexamethasone with 5HT3 antagonist with/without lorazepam and with/without proton pump inhibitors/H2 blockers [7, 12, 13]. These patients need to be protected against emesis prophylactically and throughout the entire period of risk, 3 days for HEC. More than 90% of patients on HEC develop emesis. However, with prophylactic antiemetics, the incidence reduces to <30% [10, 14, 15]. The list of drugs with highly emetogenic potential is seen in Table 1 [16]. As per the NCCN guidelines, olanzapine is one of the options for antiemetic therapy [16]. The efficacy, safety, and cost benefit of olanzapine (OLN) when compared to aprepitant (APR) in the prevention of CINV in patients receiving HEC were evaluated.

## 2. Materials and Methods

### 2.1. Type of Study

This was a prospective pilot study conducted at a regional cancer centre in South India.

### 2.2. Objectives

The objectives of the study are to evaluate the efficacy, safety, and cost benefit of olanzapine when compared to aprepitant in the prevention of CINV in patients receiving HEC.

### 2.3. Inclusion Criteria

Inclusion criteria were all adult patients aged >18 yrs and <60 yrs, chemotherapy naïve, and receiving 1st cycle of highly emetogenic chemotherapy (Table 2) and had no nausea/vomiting in the 24 hours prior to chemotherapy, serum creatinine ≤2.0 mg/dL, serum bilirubin ≤ 2.0 mg/dL, serum SGOT or SGPT less than or equal to three times the upper limit of normal, absolute neutrophil count ≥1500/mm^3^, negative urine pregnancy test in female patients of child bearing potential, no severe cognitive compromise, no known history of CNS disease/dementia, no treatment with another antipsychotic agent such as risperidone, quetiapine, clozapine, phenothiazine, or butyrophenone for 30 days prior to or during protocol therapy, no chronic administration of phenothiazine as an antipsychotic agent, no concurrent abdominal RT, no chronic alcoholism, no evidence of bowel obstruction, no known hypersensitivity to olanzapine, no known cardiac arrhythmia, congenital QT prolongation, uncontrolled CCF, or acute myocardial infarction within the previous six months, no history of uncontrolled diabetes mellitus/vestibular dysfunction, no concurrent use of opiates/quinolones, and normal serum electrolytes.

### 2.4. Exclusion Criteria

Exclusion criteria were patients aged <18 yrs and >60 yrs, comorbidities other than HEC causing emesis, patients developing intolerable side effects, or patients with mood disturbances.

### 2.5. Study Design

The study consisted of 2 groups arm A (OPD) and arm B (APD). They received the drugs and dosages as per the schedule of the 2 arms.

*Arm A (OPD)*

 D1:
   Olanzapine 10 mg PO.
   Palonosetron 0.25 mg IV.
   Dexamethasone 20 mg IV, 30–60 minutes prior to chemotherapy.
 D2–4:
   Olanzapine 5 mg PO BD.
   Oral dexamethasone 4 mg BD.

*Arm B (APD)*

 D1:
   Aprepitant 125 mg PO.
   Palonosetron 0.25 mg IV.
   Dexamethasone 12 mg IV, 30–60 minutes prior to chemotherapy.
 D2-3:
   Aprepitant 80 mg PO OD.
 D2–4:
   Oral dexamethasone 4 mg BD.

The protocol was continued with each chemotherapy cycle until discontinuation of the same regime or for a maximum of six cycles. The numbers of subjects included for this study was 50 in each of the 2 arms.

### 2.6. Assessment Procedures

Beginning with D1 of chemotherapy, until day 7, patients were asked to record daily episodes of nausea and vomiting/retching, the intensity of symptoms, and the need for rescue therapy. A Visual Analogue Score was used to assess the intensity of nausea. A coordinator contacted the patient daily to remind the patient about recording events. All toxicities were graded using the Common Toxicity Criteria (CTC). Complete remission (CR) rates (no emesis, no rescue) were analyzed at acute period (24 hours after chemotherapy), delayed period (days 2–5 post chemotherapy), and overall period (0–120 hours). For monitoring of safety, blood sugars, lipid profile, LFT, and ECG were done prior to and after each chemotherapy cycle.

### 2.7. Statistical Analysis

Calculation of median and the range was done using Microsoft excel. Data were analyzed with the Statistical Package for the Social Sciences SPSS (version 16) statistical software. *p* values < 0.05 were considered to indicate statistical significance.

## 3. Results

A total of 100 patients were studied, with 50 patients in each of the 2 arms, one receiving aprepitant and the other olanzapine. The demographic details of the study population with clinical characteristics are mentioned in Table 3. The 2 arms were well matched for age and sex parameters. The antiemetic regimens used for each of the chemotherapeutic regimens are seen in Table 4.

The mean age of the study population was 44.78 years and 43.30 years in the APD and OPD arms, respectively (*p* > 0.05). The females were the majority in both of the arms. The majority of the patients were being treated for breast cancer (Table 3).

CR was 86% for the acute period (24 hours after chemotherapy), 86% for the delayed period (days 2–5 after chemotherapy), and 80% for the overall period (0–120 hours) for 50 patients receiving the APD regimen. CR was 84% for the acute period, 88% for the delayed period, and 78% for the overall period in 50 patients receiving the OPD regimen. Patients without nausea (0, scale 0–10) were APD: 88% acute, 84% delayed, and 84% overall period, and OPD: 84% acute, 88% delayed, and 84% overall period. CR and control of nausea in subsequent chemotherapy cycles were equal to or greater than cycle 1 for both regimens. OPD was comparable to APD in the control of CINV. The differences between the two arms were not significant with respect to emesis and nausea in both acute and delayed periods (*p* > 0.05) (Table 5).

The most common treatment-related adverse events (AEs) with olanzapine were drowsiness/sedation and dizziness. Both AEs were grade 1 or 2 and were seen only in < 10% (4) patients. The drowsiness lasted for a maximum duration of 36 hrs and a minimum duration of 18 hrs in these 4 patients. In the APD arm constipation and dizziness were seen in 2 cases (<5%). Other AEs seen were asthenia/fatigue in both arms. There were no significant grade 3 or 4 toxicities.

Cost per cycle of chemotherapy in Indian Rupees (INR) was roughly 1300 for aprepitant and 50 for olanzapine tablets. Median of total cost of therapy per cycle was around 1500 INR and 270 INR for aprepitant and olanzapine group, respectively.

## 4. Discussion

Olanzapine is an atypical antipsychotic that has antiemetic properties. It binds with high affinity to several receptors involved in the CINV pathways including dopamine D1–D5, 5HT_2A_, 5HT_2C_, 5HT_3_, 5HT_6_, muscarinic, alpha-adrenergic, and histamine H_1_ receptors [17–22]. Olanzapine is cited in the NCCN and ESMO guidelines as a potential agent for breakthrough treatment of CINV [16]. Two phase II studies have demonstrated that olanzapine effectively prevents both acute and delayed chemotherapy induced nausea and vomiting (CINV) in patients receiving highly or moderately emetogenic chemotherapy [17].

A phase III randomized study of 50 patients showed comparable results with aprepitant when combined with dexamethasone and palonosetron in the prevention of CINV in HEC [18].

Dizziness was the most common toxicity observed in both study groups. The most common treatment-related adverse events (AEs) with olanzapine were drowsiness/sedation and dizziness, which were grade 1 or 2 and seen only in < 10% patients. This was comparable to the toxicities seen in the previous studies [17, 22]. In the APD arm constipation and dizziness were seen in few cases (<5%). Other AEs seen were asthenia/fatigue in both of the arms. There were no significant grade 3 or 4 toxicities. Hence, we can conclude that OLN is relatively safe as APR and can be used with relatively less toxicity profile in the Indian patients. However, future studies with larger sample and randomized trials are necessary to establish the safety of OLN in the general population.

Pharmacoeconomics is an important topic concerning cancer therapy in the developing countries. Pharmacoeconomics is a scientific discipline that compares the difference in the value of one pharmaceutical drug or drug therapy compared to another for their benefit in a particular health condition [23]. It is a branch of health economics which considers the cost (expressed in monetary terms) and effects (expressed in terms of monetary value, efficacy, or enhanced quality of life) of a pharmaceutical product and estimates the cost : benefit ratio of the drug. Pharmacoeconomic studies are helpful in optimal healthcare resource allocation in resource limited settings. A large number of patients suffering from cancer in India belong to low socioeconomic group. These patients present with advanced stage disease and delay treatment due to the high costs involved. The challenge for resource poor countries like India is to devise treatment strategies which will enable a large number of patients to avail themselves of treatment at affordable costs and obtain a substantial benefit. From the perspective of a developing country, olanzapine scores over aprepitant in terms of its cost benefit, given the equal CR benefit and tolerable toxicity profile. However, future randomized trials are required to confirm these results in a larger population.

## 5. Conclusions

In this pilot study, there was no significant difference between olanzapine and aprepitant in preventing nausea and emesis with highly emetogenic chemotherapy. Apart from drowsiness, there were no other significant side effects observed in the olanzapine arm. The cost of using olanzapine is far less compared to that of aprepitant, with potential benefits in a developing country setting. However, further randomized studies with larger sample size are required to confirm the efficacy of olanzapine and its safety profile.

## Figures and Tables

**Table 1:** Emetogenic Potential of Chemotherapeutic Agents.

Level	Agent
High emetic risk (>90% frequency of emesis)	(i) AC combination defined as either doxorubicin or epirubicin with cyclophosphamide (ii) Carmustine >250 mg/m^2^ (iii) Cisplatin ≥50 mg/m^2^ (iv) Cyclophosphamide >1500 mg/m^2^ (v) Dacarbazine (vi) Doxorubicin > 60 mg/m^2^ (vii) Epirubicin >90 mg/m^2^ (viii) Ifosfamide ≥ 10 g/m^2^ (ix) Mechlorethamine (x) Streptozocin

Moderate emetic risk (30%–90% frequency of emesis)	(i) Aldesleukin > 12–15 million international units/m^2^ (ii) Amifostine > 300 mg/m^2^ (iii) Arsenic trioxide (iv) Azacitidine (v) Bendamustine (vi) Busulfan (vii) Carboplatin (viii) Carmustine ≤ 250 mg/m^2^ (ix) Cisplatin < 50 mg/m^2^ (x) Clofarabine (xi) Cyclophosphamide ≤ 1500 mg/m^2^ (xii) Cytarabine >200 mg/m^2^ (xiii) Dactinomycin (xiv) Daunorubicin (xv) Doxorubicin ≤ 60 mg/m^2^ (xvi) Epirubicin ≤ 90 mg/m^2^ (xvii) Idarubicin (xviii) Ifosfamide < 10 g/m^2^ (xix) Interferon alfa ≥ 10 million international units/m^2^ (xx) Irinotecan (xxi) Melphalan (xxii) Methotrexate ≥ 250 mg/m^2^ (xxiii) Oxaliplatin (xxiv) Temozolomide

**Table 2:** Chemotherapy regimens included.

FEC 100/FAC	Breast

ABVD	Hodgkin's lymphoma

CHOP	Non-Hodgkin's lymphoma

CDDP paclitaxel	Head and neck

CDDP/5FU	Head and neck

IAP	Osteosarcoma

ECF	Stomach

**Table 3:** Patient demographics and baseline characteristics.

Patient characteristics	APD arm	OPD arm
Age in years (mean)	44.70 years	43.30 years
Sex		
Male, *N* (%)	15 (30%)	15 (30%)
Female, *N* (%)	35 (70%)	35 (70%)
Type of cancer		
Breast cancer	25 (50%)	26 (52%)
Lymphoma	9 (18%)	7 (14%)
Head and neck cancer	9 (18%)	10 (20%)
Osteosarcoma	5 (10%)	5 (10%)
Stomach cancer	2 (4%)	2 (4%)

**Table 4:** Antiemetic intervention for each regimen.

Chemotherapy regimen	APD arm	OPD arm
FEC 100/FAC	25	26
CHOP or ABVD	9	7
CDDP/5FU or CDDP paclitaxel	9	10
IAP	5	5
ECF	2	2

**Table 5:** Comparison of the outcomes in the APD and OPD arms.

Outcome	APD arm	OPD arm
Number of cycles received	6	6
CR rates	80%	78%
Emesis		
Acute emesis	7 (14%)	8 (16%)
Delayed emesis	7 (14%)	6 (12%)
Overall emesis	10 (20%)	11 (78%)
Nausea		
Acute nausea	6 (12%)	8 (16%)
Delayed nausea	8 (16%)	6 (12%)
Overall nausea	8 (16%)	8 (16%)
